# Supplementary material for: Optimizing a human monoclonal antibody for better neutralization of SARS-CoV-2
Source: Nat Commun. 2025 Jul 4;16:6195. doi: 10.1038/s41467-025-61472-z (PMC12227675; doi:10.1038/s41467-025-61472-z)
Supplement: Supplementary file 1 — Supplementary Information [file 41467_2025_61472_MOESM1_ESM.pdf]

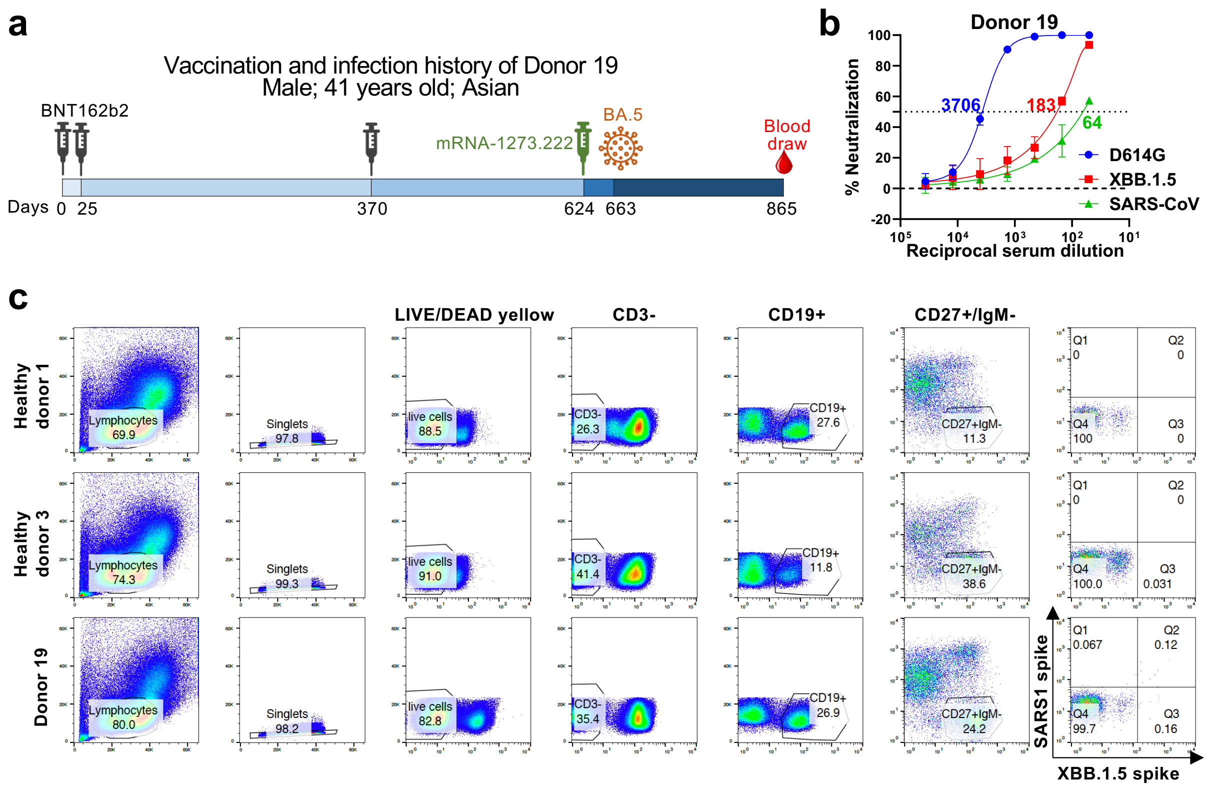

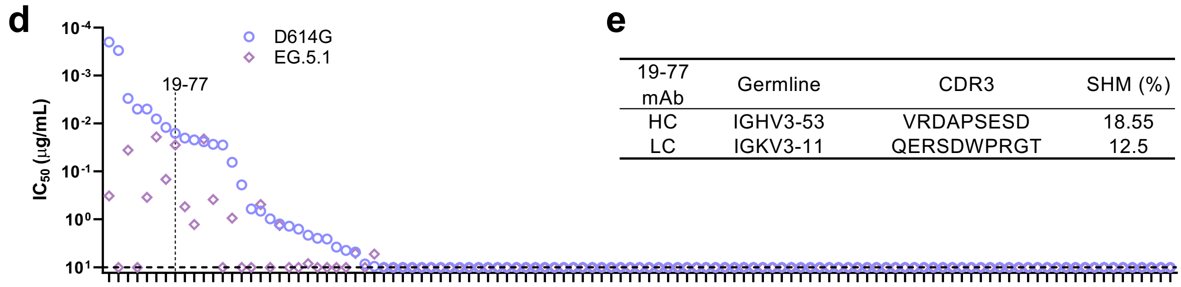

e

19-77 mAb	Germline	CDR3	SHM (%)
HC	IGHV3-53	VRDAPSESD	18.55
LC	IGKV3-11	QERSDWPRGT	12.5

Supplementary Fig. 1 | Clinical information and sorting strategy.

a. Clinical information of donor 19, whose blood sample was collected for neutralizing antibody evaluation and B cell sorting.

b. Neutralization activity of serum from donor 19 against D614G, XBB.1.5, and SARS-CoV. Neutralization ID_{50} titer against each virus is denoted. Data are shown as mean ± SEM (standard error of the mean) from technical triplicates.

c. Sorting strategy used to isolate XBB.1.5 spike and/or SARS-CoV spike specific B cells. B cells from Q2 and Q3 were collected and applied for downstream 10X Genomics analysis. Numbers in gates represent cell percentages.

d. Neutralizing IC_{50} values of the antibodies from donor 19 against D614G and EG.5.1. 19-77 is highlighted with a dotted line.

e. Germline genes, CDR3 amino acid sequences, and SHM percentages of 19-77 heavy and light chains.

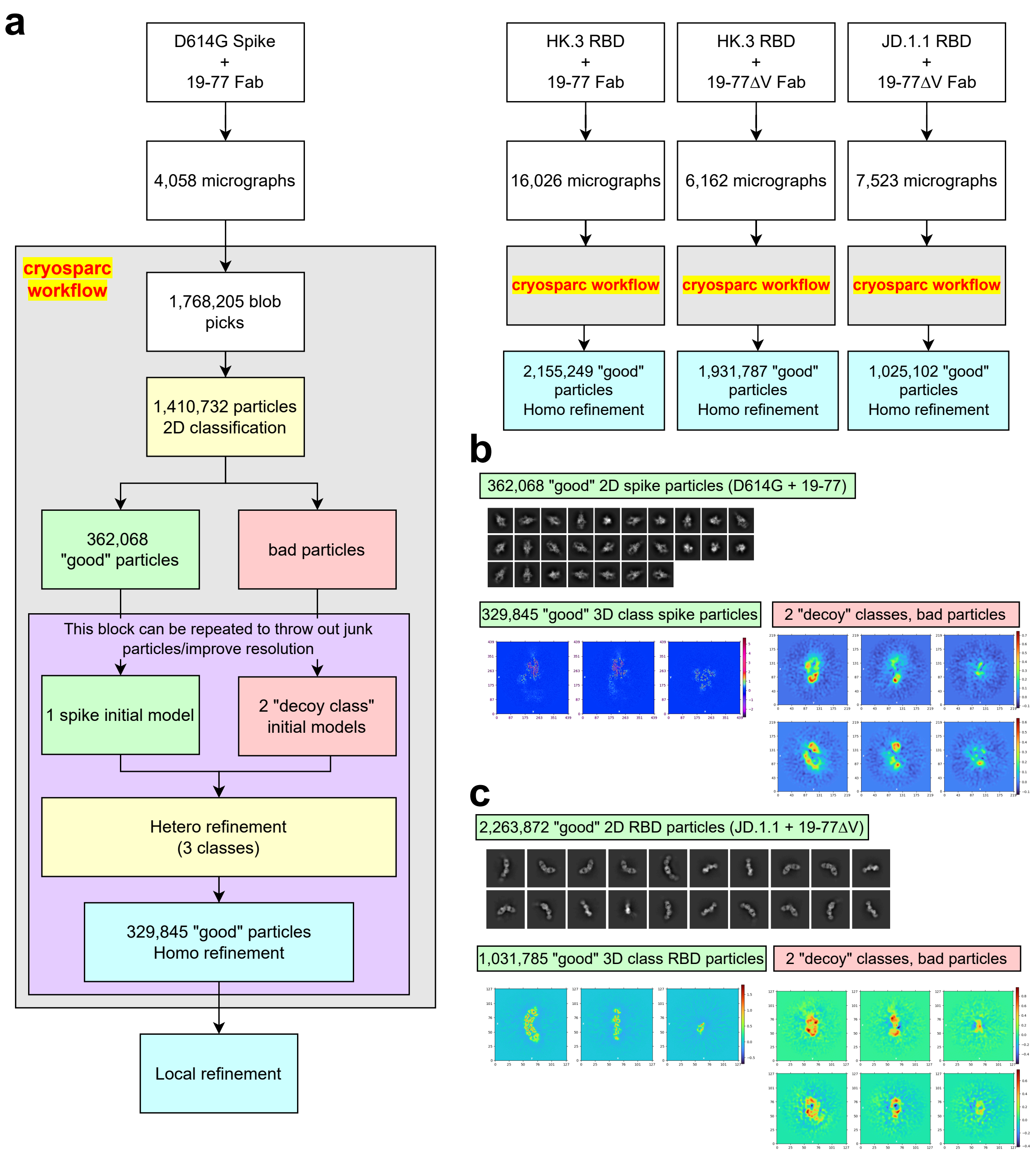

Supplementary Fig. 2 | Cryo-EM processing workflow and sample class averages

a. Cryo-EM single particle processing workflow for spike+antibody and RBD+antibody complexes. A standard cryo-EM single-particle processing pipeline was applied individually to each dataset of spike+antibody and RBD+antibody complexes.

b. Representative 2D and 3D classes of spike+antibody particles are shown using D614G and 19-77 as example.

c. Representative 2D and 3D classes of RBD+antibody particles are shown using JD.1.1 RBD and 19-77ΔV as example.

a

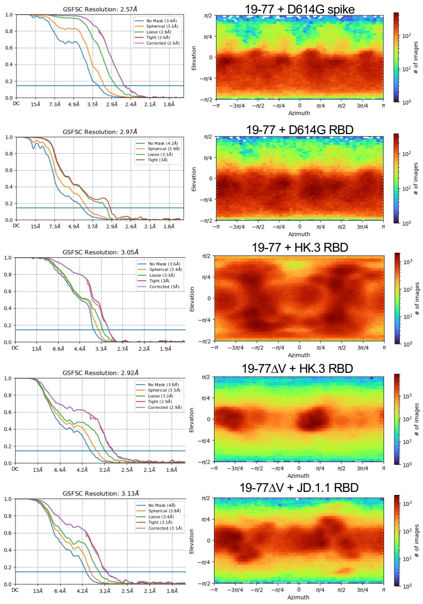

b

Cryo-EM data collection, refinement, and validation statistics for 19-77 samples

Antibody	19-77	19-77	19-77ΔV	19-77ΔV
Antigen	D614G RBD	HK.3 RBD	HK.3 RBD	JD.1.1 RBD
EMBD	EMD-45543	EMD-45544	EMD-45545	EMD-45546
PDB	9CFE	9CFF	9CFG	9CFH
**Data collection and processing**				
Magnification	105,000	105,000	105,000	105,000
Voltage (kV)	300	300	300	300
Electron exposure (e-/Å^2)	58.06	58.06	58.06	58.06
Defocus rang (μm)	-0.8 to -2.0	-0.8 to -2.0	-0.8 to -2.0	-0.8 to -2.0
Pixel size (Å/pix)	0.83	0.83	0.83	0.83
Symmetry impose	C1	C1	C1	C1
Final particle images (no.)	329,845	2,155,249	1,931,787	1,025,102
Map resolution	3.1Å	3.3Å	3.2Å	3.4Å
FSC threshold (loose mask)	0.143	0.143	0.143	0.143
**Refinement**				
Model composition				
Non-hydrogen atoms	6382	6400	6402	6394
Protein residues	416	417	417	417
Ligands				
R.m.s. deviations				
Bond lengths (Å)	0.013 (2)	0.004 (0)	0.004 (0)	0.004 (0)
Bond angles (˚)	1.928 (31)	0.625 (3)	0.744 (0)	0.747 (3)
Validation				
MolProbity score	1.32	1.74	1.47	1.25
Clashscore	0.31	4.68	5.63	2.50
Poor rotamers (%)	1.98	1.69	0.28	0.00
Ramachandran plot				
Favored (%)	92.20	95.38	97.08	96.59
Allowed (%)	6.83	4.38	2.92	3.16
Disallowed (%)	0.98	0.24	0.0	0.24
B factors (Å)				
Protein	41.52	65.72	147.56	58.15

c

X-ray diffraction data collection and refinement statistics

Antibody	19-77 Fab
Antigen	EG.5.1 RBD
PDB ID	9CAV
**Data Collection**	
Space group	$P2_1$
Cell constants	94.9 116.2 109.2<br>90 110.4 90
Resolution range (Å)	35.2 - 3.2<br>(3.1- 3.0)*
Total reflections	86848 (514)
Unique reflections	43474 (1270)
Completeness (%)	97.74 (94.91)
Redundancy	2.0 (2.0)
I/σ	3.22 (1.29)
R-merge	0.085 (0.67)
CC1/2	0.69 (0.40)
**Refinement**	
Resolution range (Å)	34.5 - 3.2
Number of complexes per asymmetric unit	2
Rwork/Rfree	0.25/0.29
Number of atoms	
Protein	8940
Ligands	0
Water	0
B-factors (Å^2)	
Protein	36.0
Ligands	-
Water	-
R.M.S deviations	
Bond lengths (Å)	0.012
Bond angles (°)	1.64
Ramachandran statistics	
Favored (%)	93.85
Allowed (%)	6.15
Outliers (%)	0

d

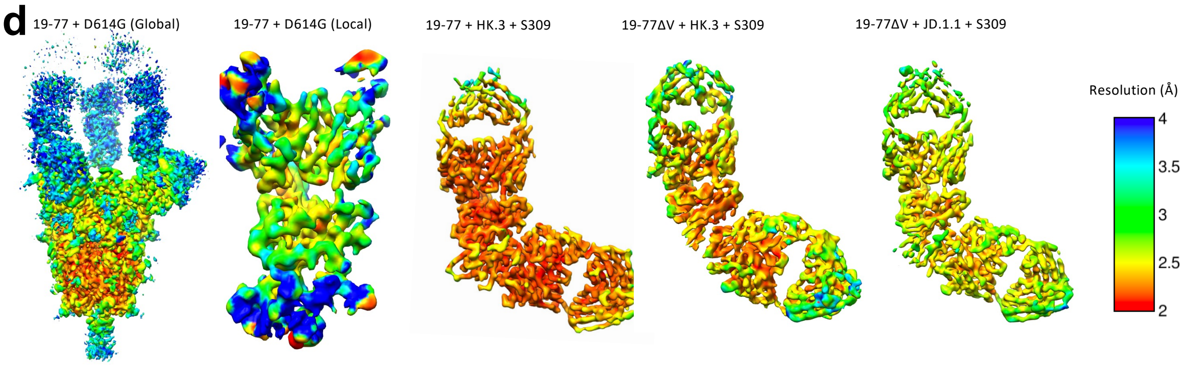

e

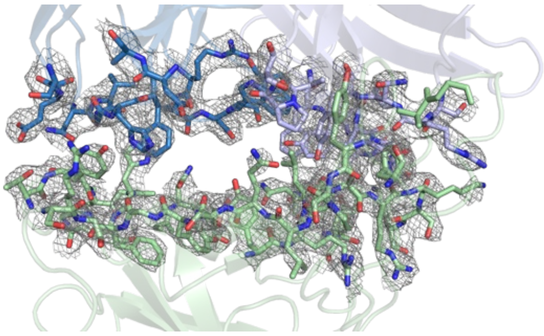

Supplementary Fig. 3 | Cryo-EM and X-ray data for mAbs 19-77 and 19-77ΔV in complex with SARS-CoV-2 spike trimers or RBDs.

a. Global refinement Fourier Shell Correction curves showing the overall resolution of the indicated complexes.

b. Cryo-EM data collection and model refinement of the indicated complexes.

c. X-ray diffraction data collection and refinement statistics.

d. Global and local resolution of Cryo-EM structures.

e. Composite omit map showing the electron density at the interface between EG5.1 RBD and 19-77 Fab. The RBD is depicted in pale green, with the Fab light and heavy chains shown in different shades of blue. The map is contoured at 1.5σ.

a

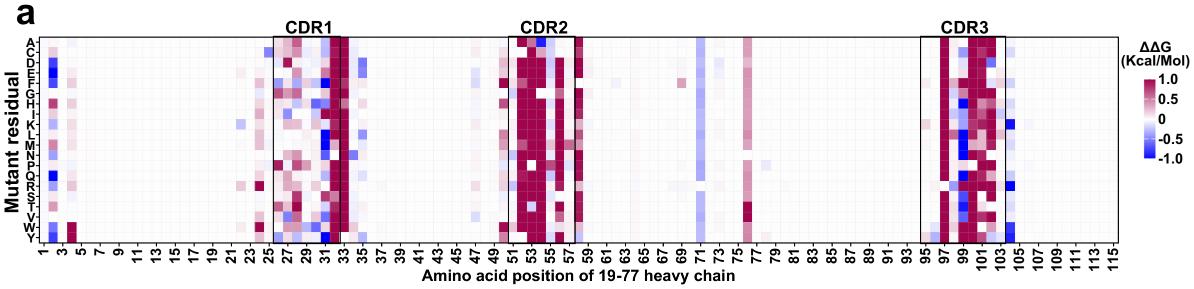

b

	IC_{50} (μg/mL)	JD.1.1	HK.3	EG.5	EG.5-A475V
	19-77	>10	0.229	0.098	>10
19-77 HC	E1A	>10	0.450	0.137	>10
	V2A	>10	0.202	0.060	>10
	S25A	>10	0.178	0.088	>10
	E26A	>10	0.570	0.281	>10
	L27A	>10	2.309	0.523	>10
	I28A	>10	0.940	0.156	>10
	S30A	>10	0.114	0.046	>10
	R31A	>10	0.210	0.069	>10
	N32A	>10	>10	>10	>10
	Y33A	>10	>10	>10	>10
	Y52A	>10	0.463	0.030	>10
	P53A	>10	0.887	0.046	>10
	G54A	>10	2.377	0.923	>10
	S56A	>10	3.750	1.092	>10
	S57A	>10	0.068	0.059	>10
	F58A	>10	>10	2.935	>10
	R71A	0.169	0.009	0.009	0.176
	N76A	>10	0.286	0.049	>10
	R97A	>10	>10	>10	>10
	D98A	>10	2.840	0.550	>10
	P100A	>10	0.241	0.584	>10
	S101A	>10	0.168	0.071	>10
	E102A	>10	0.619	0.062	>10

c

	IC_{50} (μg/mL)	JD.1.1	HK.3	EG.5	EG.5-A475V
	19-77	>10	0.229	0.098	>10
19-77 LC	I2A	>10	0.994	0.121	>10
	Q27A	>10	0.280	0.081	>10
	N28A	>10	>10	>10	>10
	I29A	>10	0.578	0.036	>10
	G30A	>10	0.294	0.066	>10
	H31A	>10	>10	1.244	>10
	F32A	>10	>10	0.440	>10
	D50A	>10	2.456	0.225	>10
	S67A	>10	0.528	0.127	>10
	E68A	>10	1.724	0.315	>10
	S92A	>10	0.135	0.074	>10
	D93A	>10	0.388	0.098	>10
	W94A	>10	>10	>10	>10
	P95A	>10	>10	0.469	>10

d

IC_{50} (μg/mL)	JD.1.1	HK.3	EG.5	EG.5-A475V
19-77	>10	0.170	0.062	>10
R71A	0.065	0.008	0.006	0.337
R71C	0.606	0.014	0.008	0.336
R71D	0.420	0.010	0.011	1.211
R71E	0.472	0.009	0.010	0.333
R71F	0.469	0.007	0.006	0.168
R71G	0.546	0.005	0.006	0.850
R71H	0.310	0.007	0.008	0.301
R71I	0.285	0.010	0.011	0.206
R71K	>10	0.048	0.030	3.394
R71L	0.095	0.008	0.013	0.115
R71M	0.241	0.008	0.012	0.195
R71N	0.324	0.010	0.006	0.321
R71P	0.259	0.010	0.007	0.82
R71Q	0.245	0.003	0.012	0.214
R71S	0.319	0.008	0.015	0.237
R71T	0.251	0.007	0.015	0.155
R71V	0.095	0.004	0.015	0.06
R71W	0.615	0.006	0.019	1.054
R71Y	0.434	0.005	0.006	0.234

<0.01 | 0.01-0.1 | 0.1-1 | 1-10 | >10

Supplementary Fig. 4 | Neutralizing IC_{50} values of 19-77 mutants.

a. Free energy change (ΔΔG) for saturation mutagenesis of the 19-77 heavy chain. Blue represents beneficial mutations, while red indicates mutations with adverse effects.

b. Neutralization IC_{50} values of 19-77 carrying the indicated mutations in the heavy chain.

c. Neutralization IC_{50} values of 19-77 carrying the indicated mutations in the light chain.

d. Neutralization IC_{50} values of 19-77 carrying various amino acid substitutions at R71 in the heavy chain.

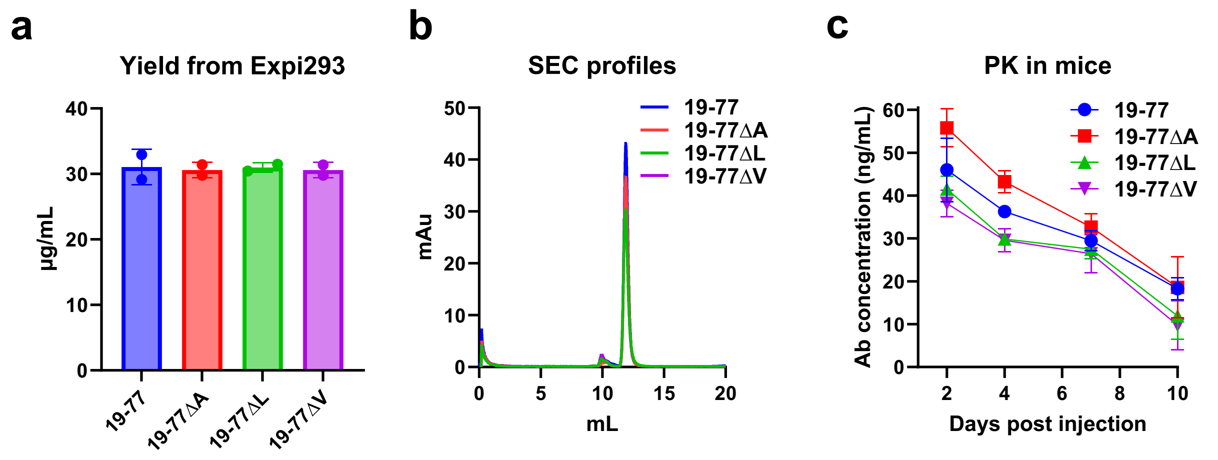

Supplementary Fig. 5 | Biological properties of 19-77.

a. Yields of 19-77 and 19-77ΔA/L/V from transiently transfected Expi293 cells at day 4 post-transfection, data show from technical duplicates.

b. Size exclusion chromatography (SEC) profiles of 19-77 and 19-77ΔA/L/V antibodies.

c. Pharmacokinetics of 19-77 and 19-77ΔA/L/V in mice over 10 days after intraperitoneal injection. Data are shown as mean ± SEM (standard error of the mean) from technical triplicates.

	Abs			Ab IC50 (ug/mL)					
				D614G	EG.5.1	BA.2.86	JN.1	HK.3	JD.1.1
VH3-53	BD56-1302	RBD class 1	WT	0.014	0.014	0.007	0.186	0.202	>10
			R71A	0.011	0.012	0.005	0.036	0.060	>10
			R71L	0.012	0.019	0.006	0.073	0.152	>10
			R71V	0.009	0.016	0.005	0.038	0.115	>10
	BD56-1854	RBD class 1	WT	0.008	0.027	0.002	0.022	0.111	10
			R71A	0.006	0.004	0.001	0.004	0.031	0.189
			R71L	0.004	0.008	0.002	0.011	0.053	0.697
			R71V	0.005	0.005	0.001	0.005	0.048	0.977
	Omi3	RBD class 1	WT	0.011	>10	0.467	>10	>10	>10
			R71A	0.009	0.668	0.021	0.685	1.505	>10
			R71L	0.009	0.991	0.048	1.324	2.232	>10
			R71V	0.005	0.172	0.020	0.331	0.912	>10
	19-79	RBD class 1	WT	0.013	0.073	0.007	>10	>10	>10
			R71A	0.011	0.042	0.002	0.116	0.165	>10
			R71L	0.016	0.160	0.006	0.484	0.582	>10
			R71V	0.011	0.062	0.003	0.124	0.162	>10
VH3-66	BD57-0129	RBD class 1	WT	0.013	0.003	0.003	>10	>10	>10
			R71A	0.013	0.003	0.002	0.028	0.107	>10
			R71L	0.015	0.005	0.003	0.064	0.132	>10
			R71V	0.013	0.003	0.002	0.035	0.090	>10
	BD515	RBD class 1	WT	0.022	>10	0.166	>10	>10	>10
			R71A	0.017	>10	0.115	0.322	>10	>10
			R71L	0.043	>10	0.157	0.913	>10	>10
			R71V	0.016	0.955	0.028	0.098	1.818	>10
VH3-74	C68.59	SD1	WT	0.048	0.108	>10	>10	0.081	0.073
			R71A	0.021	0.173	>10	>10	0.105	0.099
			R71V	0.025	0.179	>10	>10	0.085	0.120
			R71L	0.083	0.341	>10	>10	0.149	0.150
VH3-9	Omi42	RBD class 1	WT	0.020	>10	0.021	0.066	>10	>10
			R71A	0.056	>10	>10	>10	>10	>10
			R71V	0.048	>10	>10	>10	>10	>10
			R71L	0.057	>10	>10	>10	>10	>10

<0.01	0.01-0.1	0.1-1	1-10	>10

Supplementary Fig. 6 | R71/A/L/V mutations rescue the neutralizing activities of VH3-53 and VH3-66 antibodies against SARS-CoV-2 escaping variants. The figure shows neutralization IC_{50} values of the indicated antibodies and their R71A/L/V mutants.

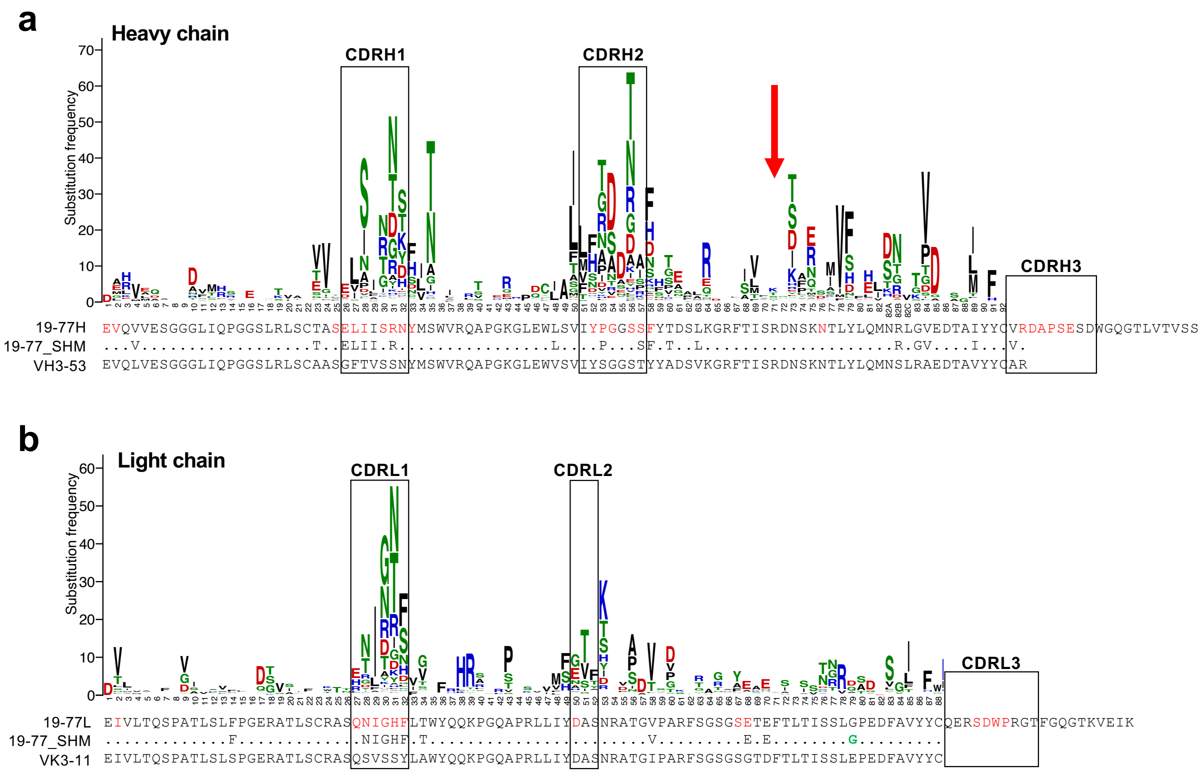

Supplementary Fig. 7 | Gene-specific substitution profile for mAb 19-77.

a. The gene-specific substitution profiles (GSSP) for IGHV3-53 and the SHMs of 19-77 heavy chain. The red residues indicate the paratope residues of 19-77 heavy chain. R71 is highlighted by a red arrow.

b. GSSP for IGKV3-11 and the SHMs of 19-77 heavy chain. The red residues indicate the paratope residues of 19-77 light chain. The green residue indicates a rare mutation in 19-77 light chain.

a

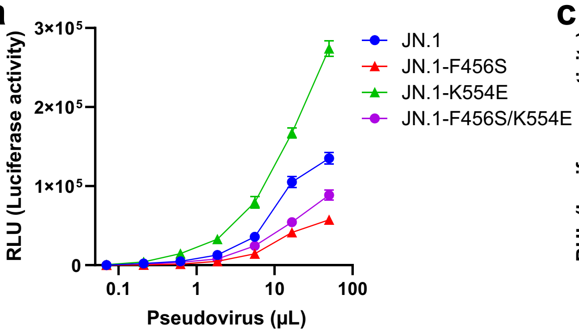

c

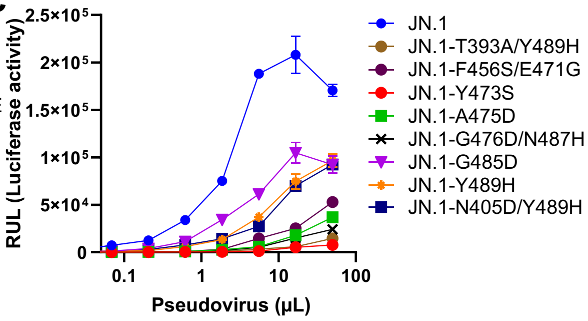

b

Neutralization (IC_{50}, μg/mL)	19-77	19-77ΔV	hACE2
JN.1	0.101	0.013	0.315
JN.1-E554K	0.104	0.009	0.167
JN.1-F456S	>10	0.675	0.304
JN.1-F456S/K554E	>10	0.424	0.695

<0.01	0.01-0.1	0.1-1	1-10	>10

d

Neutralization (IC_{50}, μg/mL)	19-77	19-77ΔA	19-77ΔL	19-77ΔV	hACE2
JN.1	0.047	0.008	0.009	0.008	0.088
JN.1-T393A/Y489H	>10	>10	>10	>10	>10
JN.1-F456S/E471G	>10	>10	>10	>10	9.039
JN.1-Y473S	>10	>10	>10	>10	>10
JN.1-A475D	>10	>10	>10	>10	>10
JN.1-G476D/N487H	>10	>10	>10	>10	>10
JN.1-G485D	>10	0.074	0.113	0.084	1.094
JN.1-Y489H	>10	0.363	0.519	0.976	2.345
JN.1-N405D/Y489H	>10	>10	>10	>10	2.967

Supplementary Fig. 8 | Infectivity and neutralization activity of 19-77ΔV escape variants in the context of VSV pseudotyped viruses.

a. Infectivity of the indicated pseudotyped escape variants selected by 19-77ΔV from authentic JN.1 in Vero-E6-TMPRSS2-T2A-ACE2 cells. Data are shown as mean ± SEM (standard error of the mean) from technical triplicates.

b. Infectivity of the indicated pseudotyped escape variants selected by 19-77ΔV from replication-competent VSV-JN.1 in Vero-E6-TMPRSS2-T2A-ACE2 cells. Data are shown as mean ± SEM from technical triplicates.

c. Neutralization IC_{50} values of 19-77, 19-77ΔV, and hACE2 against the indicated pseudotyped escape variants.

d. Neutralization IC_{50} values of 19-77, 19-77ΔA/L/V, and hACE2 against the indicated pseudotyped escape variants.
