# Supplementary material for: Optimizing a human monoclonal antibody for better neutralization of SARS-CoV-2
Source: Nat Commun. 2025 Jul 4;16:6195. doi: 10.1038/s41467-025-61472-z (PMC12227675; doi:10.1038/s41467-025-61472-z)
Supplement: Supplementary file 2 — Description of Additional Supplementary Files [file 41467_2025_61472_MOESM2_ESM.pdf]

Description of Additional Supplementary Files

Supplementary Movie 1. Molecular dynamics simulation of 19-77 and 19-77ΔA. Residues 30 and 71 are highlighted as spheres.
