# Supplementary material for: Optimizing a human monoclonal antibody for better neutralization of SARS-CoV-2
Source: Nat Commun. 2025 Jul 4;16:6195. doi: 10.1038/s41467-025-61472-z (PMC12227675; doi:10.1038/s41467-025-61472-z)
Supplement: Supplementary file 4 — Reporting Summary [file 41467_2025_61472_MOESM4_ESM.pdf]

nature portfolio

Corresponding author(s): David D. Ho

Last updated by author(s): Jun 4, 2025

Reporting Summary

Nature Portfolio wishes to improve the reproducibility of the work that we publish. This form provides structure for consistency and transparency in reporting. For further information on Nature Portfolio policies, see our Editorial Policies and the Editorial Policy Checklist.

Statistics

For all statistical analyses, confirm that the following items are present in the figure legend, table legend, main text, or Methods section.

n/a	Confirmed	
☐	☒	The exact sample size (*n*) for each experimental group/condition, given as a discrete number and unit of measurement
☐	☒	A statement on whether measurements were taken from distinct samples or whether the same sample was measured repeatedly
☒	☐	The statistical test(s) used AND whether they are one- or two-sided<br>*Only common tests should be described solely by name; describe more complex techniques in the Methods section.*
☒	☐	A description of all covariates tested
☒	☐	A description of any assumptions or corrections, such as tests of normality and adjustment for multiple comparisons
☐	☒	A full description of the statistical parameters including central tendency (e.g. means) or other basic estimates (e.g. regression coefficient) AND variation (e.g. standard deviation) or associated estimates of uncertainty (e.g. confidence intervals)
☒	☐	For null hypothesis testing, the test statistic (e.g. *F*, *t*, *r*) with confidence intervals, effect sizes, degrees of freedom and *P* value noted<br>*Give P values as exact values whenever suitable.*
☒	☐	For Bayesian analysis, information on the choice of priors and Markov chain Monte Carlo settings
☒	☐	For hierarchical and complex designs, identification of the appropriate level for tests and full reporting of outcomes
☒	☐	Estimates of effect sizes (e.g. Cohen's *d*, Pearson's *r*), indicating how they were calculated

Our web collection on statistics for biologists contains articles on many of the points above.

Software and code

Policy information about availability of computer code

Data collection	Cryo-EM data were collected using Leginon 3.4 Beta. Sequencing of memory B cell clones was performed using the Illumina NextSeq 500. Cell sorting was conducted using FACSDiva version 8.0.1.
Data analysis	Cryo-EM data was processed using cyrSPRC v2.14.2, mitonCor2, Topaz v0.2.4, 3DFSC v3.0, UCSF ChimeraX v0.93, ISOLDE v1.05, Phenix v1.18, and COOT v0.8.9.2. 10X genomics data of antibody BCRs were processed using Cell ranger v3.1.0, SONAR V1, BLAST v.2.2.25, CLUSTAO1.2.3. and USEARCH v9.2.64. Crystallization data was proceeded using XDS, AIMLESS from CCP4, and Phaser v2.8. FlowJo 11 was used for analyzing data. GraphPad Prism 10.0 and Pymol 2.5.4 were used for plotting data.

For manuscripts utilizing custom algorithms or software that are central to the research but not yet described in published literature, software must be made available to editors and reviewers. We strongly encourage code deposition in a community repository (e.g. GitHub). See the Nature Portfolio guidelines for submitting code & software for further information.

Data

Policy information about availability of data

All manuscripts must include a data availability statement. This statement should provide the following information, where applicable:

- Accession codes, unique identifiers, or web links for publicly available datasets
- A description of any restrictions on data availability
- For clinical datasets or third party data, please ensure that the statement adheres to our policy

We confirm that the antibody sequences of 19-77 and 19-79 have been deposited in GenBank under the accession number PV010127 to PV010130.

The cryo-EM and crystal structures of 19-77 in complex with various SARS-CoV-2 RBDs have been deposited in the Protein Data Bank (PDB) with the following accession codes:
1. 19-77 + SARS-CoV-2 D614G RBD (PDB: 9CFE)
2. 19-77 + HK.3 RBD (PDB: 9CFF)
3. 19-77ΔV + HK.3 RBD (PDB: 9CFG)
4. 19-77ΔV + JD.1.1 RBD (PDB: 9CFH)
5. 19-77 + EG.5.1 RBD (PDB: 9CAV)
These data are presented in Figs. 1, 2, 6, and 7, as well as in Supplementary Fig.2.

Research involving human participants, their data, or biological material

Policy information about studies with human participants or human data. See also policy information about sex, gender (identity/presentation), and sexual orientation and race, ethnicity and racism.

Reporting on sex and gender	The study included three participants: Donor 19 and two healthy donors. Donor 19 and Healthy Donor 1 are male, while Healthy Donor 2 is female. No gender-specific data were analyzed.
Reporting on race, ethnicity, or other socially relevant groupings	Donor 19 is Asian, healthy donor 1 is non-Hispanic White, and healthy donor 2 is Black/African American. No additional data on race, ethnicity, or social grouping were collected or considered in the analysis.
Population characteristics	Donor 19 is a 41-year-old Asian male who received four doses of a COVID-19 mRNA vaccine and experienced a BA.5 infection. The other donors had no history of COVID-19 mRNA vaccination or infection. No additional demographic, genotypic, or medical treatment data were reported.
Recruitment	We enrolled donor 19, who had received multiple doses of COVID-19 mRNA vaccines and experienced a COVID-19 infection. Their serum potentially exhibits potent and broad neutralizing activity against SARS-CoV-2. The purpose of this enrollment was to obtain peripheral blood mononuclear cells for identifying monoclonal antibodies against SARS-CoV-2 for therapeutic and prophylactic applications. The other healthy donors were recruited as controls for the project.
Ethics oversight	The participants provided written informed consent, and the serum collection was performed under protocols reviewed and approved by the Institutional Review Board of Columbia University.

Note that full information on the approval of the study protocol must also be provided in the manuscript.

Field-specific reporting

Please select the one below that is the best fit for your research. If you are not sure, read the appropriate sections before making your selection.

☒ Life sciences ☐ Behavioural & social sciences ☐ Ecological, evolutionary & environmental sciences

For a reference copy of the document with all sections, see nature.com/documents/nr-reporting-summary-flat.pdf

Life sciences study design

All studies must disclose on these points even when the disclosure is negative.

Sample size	Three participants were selected for our study, with a particular focus on donor 19 due to their serum's potentially robust neutralizing activity, attributed to multiple COVID-19 mRNA vaccine boosters and a history of SARS-CoV-2 infection. The suitability of donor 19 for the study was confirmed through neutralization assays, as shown in Supplementary Fig. 1b.
Data exclusions	None
Replication	All experiments described in the manuscript were performed and verified in multiple replicates, as detailed in their methods or figure legends.
Randomization	Antibody screening from antigen-positive B cells was conducted using the neutralization assays in Supplementary Fig. 1 without any bias in selection. The selection criteria for potential antibody candidates were determined solely by the activity of individual antibodies.
Blinding	Blinded scoring of authentic SARS-CoV-2 virus neutralization in Fig. 7a was conducted, and the average scores, determined by evaluating cytopathic effects, were converted to neutralization percentages. The results are presented as mean ± SEM.

Reporting for specific materials, systems and methods

We require information from authors about some types of materials, experimental systems and methods used in many studies. Here, indicate whether each material, system or method listed is relevant to your study. If you are not sure if a list item applies to your research, read the appropriate section before selecting a response.

Materials & experimental systems

n/a	Involved in the study	
☐	☒	Antibodies
☐	☒	Eukaryotic cell lines
☒	☐	Palaeontology and archaeology
☐	☒	Animals and other organisms
☒	☐	Clinical data
☒	☐	Dual use research of concern
☒	☐	Plants

Methods

n/a	Involved in the study	
☒	☐	ChIP-seq
☐	☒	Flow cytometry
☒	☐	MRI-based neuroimaging

Antibodies

Antibodies used

For antigen-specific B cell sorting and single-cell BCR sequencing, the following antibodies were used: anti-human CD3 PerCP-Cy5.5 (BioLegend, Cat. 344808, Clone: SK7, Lot. B440848), anti-human CD19 APC/Cyanine 7 (BioLegend, Cat. 302218, Clone: HIB19, Lot. B361547), anti-human CD27 APC (BioLegend, Cat. 356410, Clone: M-T271, Lot. B324051), anti-human IgM FITC (BioLegend, Cat. 314506, Clone: MHM-88, Lot. B268793), and anti-His PE/Dazzle™ 594 (BioLegend, Cat. 362631, Clone: J095G46, Lot. B379900).For epitope mapping by ELISA, anti-human IgG (Jackson ImmunoResearch, Cat. 109-035-003, Polyclonal, Lot. 146269) was used. Monoclonal antibodies (19-77, 19-77 mutants, 19-79, P4J15, VYD222, BD56-1302, BD56-1854, Omi3, BD57-0129, BD515, C68.59, Omi42) were expressed and purified in-house, as previously described in Liu et al., 2020 (Nature), and in the Methods section of this manuscript.

Validation

All validations are available on the commercial website under the validation sheet link for each cataloged item.
1. anti-human CD3 PerCP-Cy5.5(Biolegend,Cat.344808), https://www.biolegend.com/en-gb/products/percp-cyanine5-5-anti-human-cd3-antibody-6932;
2. anti-human CD19 APC/Cyanine 7(Biolegend,Cat. 302218), https://www.biolegend.com/en-gb/products/apc-cyanine7-anti-human-cd19-antibody-1910;
3. anti-human CD27 APC (Biolegend,Cat. 356410), https://www.biolegend.com/en-gb/products/apc-anti-human-cd27-antibody-8467;
4. anti-human IgM FITC (Biolegend,Cat. 314506), https://www.biolegend.com/en-gb/products/fitc-anti-human-igm-antibody-2880;
5. anti-His PE/DazzleTM 594 (Biolegend,Cat.362631), https://www.biolegend.com/en-gb/products/pe-dazzle-594-anti-his-tag-antibody-22684;
6. anti-human IgG(Jackson ImmunoResearch, Cat.109-035-003), https://www.biocompare.com/9776-Antibodies/5606971-Peroxidase-AffiniPure-Goat-Anti-Human-IgG-H-L/;

All antibodies have been validated in previous studies involving the neutralization of SARS-CoV-2. Specifically, P4J15 was newly produced and tested prior to its use in this study, confirming results consistent with those of the original publication (Fenwick et al., 2023, J Infect). VYD222 was tested in Wang et al., 2024, NEJM. Additionally, BD56-1302, BD56-1854, Omi3, BD57-0129, BD515, C68.59, and Omi42 were tested in Wang et al., 2023, Nature.

Eukaryotic cell lines

Policy information about cell lines and Sex and Gender in Research

Cell line source(s)	HEK293T cells (Cat# CRL-3216) and Vero-E6 cells (Cat# CRL-1586) were sourced from ATCC. Expi293 cells (Cat# A14527) were sourced from Thermo Fisher.
Authentication	Cells were purchased from authenticated vendors, and their morphology was visually confirmed prior to use.
Mycoplasma contamination	The cell lines were tested and confirmed to be mycoplasma-negative.
Commonly misidentified lines (See ICLAC register)	No commonly misidentified cell lines were used in this study.

Animals and other research organisms

Policy information about studies involving animals; ARRIVE guidelines recommended for reporting animal research, and Sex and Gender in Research

Laboratory animals	6-8 wk old BALB/c mice (Strain #:000651) were purchased from The Jackson Laboratory (Bar Harbor ME). Mice were housed in groups of 4-5 per cage. There was active photo-period of 12h on/off light/dark cycle. Ambient animal room temperature was maintained at 72degF ± 3deg. Room humidity was 50%, and maintained within ± 10%.
Wild animals	No wild animals were used.
Reporting on sex	Female mice were used due to prior literature on their permissiveness to agents tested.
Field-collected samples	No field collected samples were used.

Ethics oversight

All animal experiments were carried out in strict accordance with the Policy on Humane Care and Use of Laboratory Animals of the United States Public Health Service. The protocol was approved by the Institutional Animal Care and Use Committee (IACUC) at The Columbia University (Animal Welfare Assurance no. D16-00003). Retrobulbular blood collections for antibody pharmacokinetics were conducted using approved anesthesia and/analgesics under the protocol. Mice were euthanized with CO_2, with every effort made to minimize suffering following the guidelines.

Note that full information on the approval of the study protocol must also be provided in the manuscript.

Plants

Seed stocks

Report on the source of all seed stocks or other plant material used. If applicable, state the seed stock centre and catalogue number. If plant specimens were collected from the field, describe the collection location, date and sampling procedures.

Novel plant genotypes

Describe the methods by which all novel plant genotypes were produced. This includes those generated by transgenic approaches, gene editing, chemical/radiation-based mutagenesis and hybridization. For transgenic lines, describe the transformation method, the number of independent lines analyzed and the generation upon which experiments were performed. For gene-edited lines, describe the editor used, the endogenous sequence targeted for editing, the targeting guide RNA sequence (if applicable) and how the editor was applied.

Authentication

Describe any authentication procedures for each seed stock used or novel genotype generated. Describe any experiments used to assess the effect of a mutation and, where applicable, how potential secondary effects (e.g. second site T-DNA insertions, mosiacism, off-target gene editing) were examined.

Flow Cytometry

Plots

Confirm that:

- [x] The axis labels state the marker and fluorochrome used (e.g. CD4-FITC).
- [x] The axis scales are clearly visible. Include numbers along axes only for bottom left plot of group (a 'group' is an analysis of identical markers).
- [x] All plots are contour plots with outliers or pseudocolor plots.
- [x] A numerical value for number of cells or percentage (with statistics) is provided.

Methodology

Sample preparation

Peripheral blood mononuclear cells from Donor 19 and two healthy donors were stained using the LIVE/DEAD Fixable Yellow Dead Cell Stain Kit (Invitrogen) at ambient temperature for 20 minutes. The cells were then washed with RPMI 1640 complete medium [RPMI 1640 supplemented with 10% fetal bovine serum (FBS) and penicillin/streptomycin (P/S) at 100 U/mL] and incubated with 10 μg/mL XBB.1.5 S2P protein and 10 μg/mL biotinylated SARS-CoV S2P at 4°C for 45 minutes. Following incubation, the cells were washed again and labeled with a cocktail of flow cytometry and Hashtag antibodies, including CD3 PerCP-Cy5.5, CD19 APC/Cyanine 7, CD27 APC, IgM FITC, anti-His PE/DazzleTM 594, Streptavidin BV421, and human Hashtag 3 at 4°C for 1 hour. After staining, the cells were washed, resuspended in RPMI 1640 complete medium, and sorted by flow cytometry for SARS-CoV and/or XBB.1.5 S2P trimer-specific memory B cells (CD3− CD19+ CD27+ IgM− antigen+ live single lymphocytes).

Instrument

BD FACSAriaII (P695001499)

Software

FACSDiva version 8.0.1

Cell population abundance

SARS-CoV and/or SARS-CoV-2 spike protein-positive cells were purified from the PBMCs of Donor 19 using the gating strategy outlined below. Purified trimer-positive memory B cells from Donor 19 were compared to those from two healthy donors (native controls), as shown in Supplementary Fig. 1c.

Gating strategy

As shown in Extended Data Fig. 1c, sorting of PBMCs was performed in the same manner for all samples, including healthy donors. The gating process is summarized as follows: All PBMCs were initially gated using FSC-A and SSC-A to define lymphocyte populations. Lymphocytes were then gated using 'Trigger Pulse Width' and FSC-A to isolate singlets. Singlets were further gated based on fluorescence from the LIVE/DEAD™ Fixable Yellow Dead Cell Stain Kit to identify live cells. Next, the CD3 negative population was selected by gating the SSC-A versus CD3-PerCP-Cy5.5-stained population. B cells were identified within the negative population by selecting IgM negative cells, followed by CD27 positive cells on their respective fluorescence channels. Finally, a subset of CD19 positive cells was selected for SARS-CoV and/or XBB.1.5 S2P trimer-specific memory B cells (CD3-/CD19+/IgM−/ CD27+/Antigen+/ Live single lymphocytes).

- [x] Tick this box to confirm that a figure exemplifying the gating strategy is provided in the Supplementary Information.
